# Complete Genome Sequence of Campylobacter jejuni Strain 12567, a Livestock-Associated Clade Representative

**DOI:** 10.1128/genomeA.00513-18

**Published:** 2018-06-14

**Authors:** J. C. Sacher, E. Yee, C. M. Szymanski, W. G. Miller

**Affiliations:** a Department of Biological Sciences, University of Alberta, Edmonton, Alberta, Canada; b Produce Safety and Microbiology Research Unit, Agricultural Research Service, U.S. Department of Agriculture, Albany, California, USA; c Department of Microbiology and Complex Carbohydrate Research Center, University of Georgia, Athens, Georgia, USA

## Abstract

We report here the complete genome sequence of Campylobacter jejuni strain 12567, a member of a C. jejuni livestock-associated clade that expresses glycoconjugates associated with improved gastrointestinal tract persistence.

## GENOME ANNOUNCEMENT

Campylobacter jejuni, a human gastrointestinal pathogen causing diarrheal disease (1), naturally colonizes chickens, so human infections commonly arise from the consumption and mishandling of contaminated poultry products (2). Expression of highly variable, glycosylated surface structures is important for C. jejuni poultry persistence, and further understanding of the mechanisms behind their presentation can inform strategies for reducing bacterial burden (3, 4).

C. jejuni strain 12567 is a poultry-derived livestock-associated clade representative (3), a poorly studied isolate whose glycoconjugates have been disproportionately well characterized (5, 6). The capsular polysaccharide (CPS) modification *O*-methyl phosphoramidate (MeOPN), which mediates C. jejuni*-*phage interactions (7), was found on the strain 12567 CPS (6). Strain 12567 also expresses precursors for the flagellar legionaminic acid modifications Leg5Am7Ac and Leg5AmNMe7Ac, which are thought to assist in chicken colonization (3). Binding of 12567 by a phage protein specific for the flagellar pseudaminic acid derivative Pse5Ac7Am suggests that 12567 also synthesizes this glycan (3, 8, 9). The 12567 genome sequence therefore provides a resource for studying different C. jejuni glycoconjugate expression mechanisms.

We present here the complete genome sequence of C. jejuni strain 12567. The Illumina MiSeq and PacBio RS next-generation sequencing platforms were used to complete the genome. Assembly of the MiSeq reads generated a draft genome of 427 contigs; closure of the genome, especially across the flagellar modification, lipooligosaccharide, and CPS loci, required PacBio sequencing. Illumina MiSeq reads (920-fold coverage) were used to validate all base calls and determine the variability of each poly(G) tract. The final coverage across the genome was 1,318-fold. Strain 12567 has a circular genome of 1,706 kbp with an average GC content of 30.4%. Protein-, rRNA-, and tRNA-encoding genes were identified as described (10). The genome contains 1,628 putative protein-coding genes and 49 pseudogenes.

Examination of the 12567 CPS locus showed gene content almost identical to the reference strain, C. jejuni NCTC 11168, with the only difference being three MeOPN transferase genes (CJ12567_1407, CJ12567_1408, and CJ12567_1409) in strain 12567 compared to two (*cj1421c* and *cj1422c*) in strain 11168. The flagellar glycosylation loci of strains 12567 and 11168 are also similar, although only 12567 encodes functional copies of an aminoglycoside N3ʹ-acetyltransferase and the methyltransferase *ptmH*, both of which are pseudogenes in strain 11168. The presence of *ptmH* in 12567 provides a possible explanation for why this strain makes Leg5AmNMe7Ac (3), which has been shown to require *ptmH* in Campylobacter coli VC167 (11). Strain 12567 also exhibits differences in motility-associated factor (*maf*) gene content compared to that of strain 11168. Although 12567 encodes the same number of poly(G) tract-containing *maf* genes as 11168, the position of the *maf* genes *maf1*, *maf3*, *maf5*, and *maf6* within the flagellar locus and the presence/absence of phase-variable poly(G) tracts within these genes differs between the two strains.

The genome sequence of strain 12567 provides genetic information to complement the phenotypic characterization of its CPS and flagellar glycans. While this strain exhibits many similarities to 11168, also a livestock-associated strain, the differences in gene content at two biologically relevant glycoconjugate biosynthesis loci provide a new understanding of C. jejuni glycobiology.

### Accession number(s).

The complete genome sequence of C. jejuni strain 12567 has been deposited in GenBank under the accession number CP028909.
